# Chronic rhinosinusitis with nasal polyps (CRSwNP) symptom verbal response scales: content validity testing for use in adults with CRSwNP

**DOI:** 10.1186/s41687-024-00827-4

**Published:** 2024-12-20

**Authors:** Tom Keeley, Nina Gaw, Waseem Ahmed, Rafael Alfonso-Cristancho, Ana R. Sousa, Katie Forde, Rosie Sharp, Sophie Whyman, Adam Gater

**Affiliations:** 1 Patient Centered Outcomes, GSK, London, UK; 2 Global Data Generations, GSK, London, UK; 3 Global Data Generations, GSK, Collegeville, PA USA; 4 Clinical Sciences, Respiratory and Immunology Research Unit, GSK, London, UK; 5 Patient-Centered Outcomes, Adelphi Values, Bollington, Cheshire, UK

**Keywords:** Patient-reported outcome measures, Content validity, Verbal response scale, Chronic rhinosinusitis, Nasal polyps, Psychometrics

## Abstract

**Background:**

PRO measures of symptoms in clinical trials have historically utilized visual and numerical scales but verbal descriptors may make it easier for patients to clearly differentiate between response options. This study assessed content validity and meaningful change in five verbal response scales (VRSs) used to assess chronic rhinosinusitis with nasal polyp (CRSwNP) symptom severity.

**Methodology:**

This qualitative, semi-structured interview study recruited adults from the US, Germany, and China with confirmed moderate-to-severe CRSwNP. Interviews included a concept elicitation section, where participants were asked about their experience of living with CRSwNP including symptoms and health-related quality-of-life impacts, and a cognitive debriefing section, where participants were debriefed and participant understanding and real-life relevance of the CRSwNP symptom VRS content were assessed. Interview transcripts were qualitatively analyzed using thematic analysis methods.

**Results:**

Among the 24 participants interviewed, the most frequently reported CRSwNP symptoms were nasal obstruction, runny nose, mucus in the throat, loss of smell and facial pain/pressure. Participants demonstrated good understanding of the CRSwNP symptom VRS instructions, items, recall period, and response options. The five CRSwNP symptom VRS items were relevant to the majority of participants’ experience of CRSwNP. At the item level, a one-category within-person improvement was the level most frequently reported by participants to be a meaningful change.

**Conclusion:**

The CRSwNP symptom VRSs assess relevant and bothersome symptoms experienced by patients with moderate-to-severe CRSwNP, supporting content validity of this measure. The findings of this study provided preliminary insights into meaningful change in the VRS. Further quantitative assessment of meaningful change is needed, and psychometric evaluation of the CRSwNP symptom VRSs will be required to evaluate their appropriateness for assessment of clinical trial endpoints in patients with CRSwNP.

**Supplementary Information:**

The online version contains supplementary material available at 10.1186/s41687-024-00827-4.

## Background

Chronic rhinosinusitis with nasal polyps (CRSwNP) is an inflammatory disease of the sinuses that is estimated to affect 1–4% of the global population; core symptoms include nasal obstruction, watery rhinorrhea from the nose (anterior), postnasal drainage (posterior), a temporary or permanently decreased sense of smell (anosmia), and facial pain [1, 2]. These symptoms can have a significant negative impact on patients’ health-related quality of life (HRQoL) [2]. Current standard-of-care treatment aims to treat the underlying inflammation and symptoms of CRSwNP, while also improving HRQoL, and includes saline irrigation, topical intranasal corticosteroids, and systemic corticosteroids (SCS), in addition to functional endoscopic sinus surgery [2, 3]. However, disease recurrence after surgery and high symptom burden can lead to patients requiring repeat courses of SCS, putting them at risk of adverse effects and alternative treatment options are needed to address the high clinical and economic burden associated with CRSwNP [4].

Patient-reported outcome (PRO) measures are used in clinical trials to assess efficacy and ensure that treatments under development address the needs of the population that they are intended to treat [5]. Recent clinical trials for new treatments for CRSwNP have used changes from baseline in endoscopic nasal polyp scores (centrally read) and a PRO measure for nasal obstruction to assess the primary endpoints [6–8]. One such PRO measure is the CRSwNP symptom visual analog scale (VAS) score; this measure includes five items assessing nasal obstruction, nasal discharge, mucus in the throat, loss of smell, and facial pain or pressure, as well as one further VAS assessing overall symptom severity. Patients are asked to rate their symptom severity using the CRSwNP VAS measure for the period of the past 24 h, on a scale of 0 (none) to 10 (as bad as you can imagine) [6, 9, 10].

However, verbal descriptors within PRO measures can make it easier for patients to clearly differentiate between response options. Recent guidance from the US Food and Drug Administration (FDA) has recommended that co-primary endpoints for clinical trials include PRO measures for the assessment of nasal congestion using a verbal response scale (VRS), with four levels (often scored from 0 to 3) that can be clearly defined and represent clinically meaningful and distinct response categories such as ‘no symptoms’, ‘mild symptoms’, ‘moderate symptoms’, and ‘severe symptoms’ [11]. In addition to this, the FDA recommends that secondary endpoints include assessments using PRO scores that are relevant and important to patients and are also rated on a four-level VRS [11].

Here, we report findings from qualitative interviews with participants with moderate-to-severe CRSwNP. The aim was to evaluate the content validity of five VRSs assessing CRSwNP symptom severity (adapted from closely aligned previously utilized CRSwNP symptom VAS) [6, 9, 10], as well as the content validity of the Patient Global Impression of Severity (PGI-S) and Patient Global Impression of Change (PGI-C) measures in this population. We assessed the relevance of the CRSwNP symptom VRS items to participants’ experience of CRSwNP qualitatively explored definitions of meaningful change for all measures.

## Methods

### Study design and data collection procedures

This study was a qualitative, semi-structured interview study enrolling participants in the US, Germany, and China (GSK study ID: 218108). The sample size was selected with the aim of achieving sample diversity while also enabling the identification of any problems with the CRSwNP symptom VRS items and facilitating the exploration of meaningful change [12]. Quota sampling, a non-probability sampling technique whereby participants with predefined characteristics are selected [13], was employed to obtain insights from a diverse population of participants with a range of socio-demographic characteristics (Supplementary Table 1). Participants from the US were recruited via social media advertising, participants from Germany were recruited from a pre-existing patient database, and participants from China were recruited via healthcare professional referrals. The study aimed to recruit a total of 24 adults (US, *n* = 12; Germany, *n* = 6; China, *n* = 6) with a diagnosis of CRSwNP; the participant recruitment procedure is summarized in Supplementary Fig. 1.

Country-specific informed consent forms detailing the study purpose, roles and responsibilities of participants while enrolled in the study, relevant data protection legislations, and compensation for participating in the interview were completed by study participants. Individuals in the US and Germany provided consent to have their patient screener form and redacted diagnostic evidence (see *Study participants* subsection) shared with Adelphi Values for the purpose of verifying their eligibility. For data protection purposes, individuals in China provided consent to participate in an interview, but no patient identifiable information for the Chinese participants was shared with the project team; the recruitment agency reviewed participants’ evidence of CRSwNP diagnosis and patient screener forms and confirmed participants’ eligibility with Adelphi Values. Socio-demographic and clinical characteristics data were collected via demographics and patient screener forms, respectively.

### Study participants

Participants included in the study were ≥ 18 years of age with confirmed recurrent CRSwNP (by diagnosis on electronic health records), who had previous nasal surgery to remove polyps or ≥ 3 consecutive days of SCS use for CRSwNP in the last 2 years (but not in the previous 28 days), and who reported moderate-to-severe nasal obstruction in the previous 24 h and additional CRSwNP symptoms within the previous 3 months. Participants were excluded from the study if they had a diagnosis of cystic fibrosis, sinus infection/cold/COVID-19 symptoms (or a positive COVID-19 test) in the previous 2 weeks, or were enrolled in a clinical trial for CRSwNP in the previous 6 months.

The study was conducted according to the principles outlined in the Declaration of Helsinki. Additionally, all data was handled in accordance with the European General Data Protection Regulation (GDPR), and data from China was handled in accordance with the Personal Information Protection Law (PIPL) and Data Security Law (DSL). All Adelphi Values study team members involved in this project received tailored training in relation to these guidelines and regulations. Prior to the collection or processing of participant data, ethical approval was obtained from Salus Institutional Review Board (IRB), a centralized IRB that provides ethical oversight to research conducted globally for multi-country studies (Salus IRB: C218108 GK9381A). Each participant provided written and verbal consent via the informed consent form before their interview was conducted.

### Study assessments

The five CRSwNP symptom VRS items evaluated the core CRSwNP symptoms of nasal obstruction, nasal discharge, mucus in the throat, loss of smell, and facial pain/pressure, per US FDA guidance. The items ask participants to rate the presence and severity of these symptoms over the previous 24 h as either ‘no symptoms’, ‘mild symptoms’, ‘moderate symptoms’, or ‘severe symptoms’, and convert responses into a score (0 [no symptoms] to 3 [severe symptoms]). The specific symptom questions asked are: ‘Please rate your nasal obstruction at its worst over the previous 24 h’, ‘Please rate your runny nose at its worst over the previous 24 h’, ‘Please rate your feeling of mucus in the throat at its worst over the previous 24 hours’ ‘Please rate your loss of smell at its worst over the previous 24 hours’ and ‘Please rate your facial pain or pressure at its worst over the previous 24 hours’.

The PGI-S and PGI-C were evaluated alongside the symptom VRSs so that they could be used as anchor measures in a future psychometric analysis. The PGI-S and PGI-C are single-item measures to capture a patient’s perception of their symptom severity (PGI-S) and change in severity (PGI-C) since the start of a study which are commonly used in drug development and evaluation [14]. The specific PGI-S and PGI-C used in this study used five-point ordinal scales. For the PGI-S, participants were asked: ‘Please choose the response below that best describes the overall severity of your nasal polyps symptoms over the past 4 weeks (no symptoms, mild, moderate, severe, very severe)’. For the PGI-C, participants were asked: ‘Please choose the response below that best describes the overall change in your nasal polyps symptoms compared to when you started the study (much better, a little better, no change, a little worse, much worse)’. As patients were excluded from this study if they had participated in a clinical trial for CRSwNP in the previous 6 months, the PGI-C measure was posed as a hypothetical question.

### Interview procedure

Each participant took part in a 60-minute, one-on-one interview with a trained qualitative researcher; all interviews were conducted in the participant’s local language. Translated versions of the measures, which had been linguistically validated and cognitively tested in accordance with the Professional Society for Health Economics and Outcomes Research (ISPOR) Translation and Linguistic Validation Task Force recommendations, were used (German for German participants, and Mandarin Chinese for Chinese participants) [15]. An interview guide was used to ensure that all topics of interest were discussed. This was designed to be used as a guide, and interviewers were flexible in the order of questioning, following the lead of the participant and asking appropriate questions when topics of interest arose.

Prior to each interview, the interviewer reviewed the participant’s completed study documents (patient screener, informed consent, and demographics forms) to understand the participant’s background and help contextualize interview responses. In addition, the interviewer obtained verbal consent at the beginning of each interview to confirm the participant’s agreement to take part in the interview and to have the interview audio-recorded.

The interview comprised of: (1) a concept elicitation section, where broad open-ended questions followed by probes to explore concepts of interest were used to confirm symptoms of CRSwNP (type, frequency, duration, and severity) and impact on daily living activities, and (2) a cognitive debriefing section, where detailed interview materials were used to assess the content validity of the CRSwNP symptom VRS items, PGI-S, and PGI-C by identifying participant understanding of these measures, the relevance of the CRSwNP symptom VRS items for each participant, and the within-person improvement in score category that would constitute a meaningful change for all measures. For each item, interviewers asked participants what level of improvement would be meaningful or important to them, based on their initial severity rating provided in response to the item, and why. Participants who had experienced the symptom but not over the previous 24 h were asked to rate the highest severity of the symptom that they had ever experienced and were asked what they would consider a meaningful improvement based on this hypothetical response. Concept elicitation questioning was conducted before participants saw the interview materials (i.e., screenshots of the CRSwNP symptom VRS, CRSwNP symptom VAS, PGI-S, and PGI-C) to ensure participants’ responses were not biased by the content of these measures.

### Data analysis

Socio-demographic and clinical characteristics were summarized using totals (N values) and percentages.

All interviews were transcribed for the purpose of qualitative analysis. Verbatim German and Chinese interview transcripts were translated into US English for data analysis. Quotes were sorted by domain using thematic analysis methods and facilitated via the use of ATLAS.ti software [16]. Each transcript was assessed and participant comments pertaining to the main research questions were highlighted by assigning relevant ‘codes’. The axial coding process enabled relationships between concepts to be explored. Two members of the coding team separately coded the first two transcripts, and a coding scheme was created based on the analysis of these transcripts, which was used to code the remainder of the transcripts. Quality control checks were performed by the project lead on the analysis of certain transcripts selected at random. Prior to coding each transcript, each coder read through the entire transcript to better understand the participant’s context and experience. Throughout the analysis process, the study team met on a regular basis to resolve any discrepancies through discussion and a consensus-building process. The code list was updated iteratively and organically throughout the analysis, and previously coded transcripts were revisited and reviewed to identify any instances where the new codes may apply. Following review and coding of all transcripts, all coded data, themes, and supporting quotes were tabulated in ATLAS.ti to support further analysis and summarizing of data for reporting purposes. Further details of interviewer training are given in Supplementary Methods. Formal subgroup analyses were not performed, as no notable country differences were anticipated based on prior research. However, any issues associated with understanding that were unique to participants from certain countries were highlighted by analysts where relevant.

Saturation, the point at which no new insights are likely to be obtained from analysis of further interviews [12], was assessed post hoc on the data obtained from the concept elicitation portion of the interviews to ensure that all important concepts emerged in the sample. To evaluate whether saturation had been achieved, transcripts were split into 5 sets of four or five participants according to the order in which they were conducted. The concepts that emerged from the first set of participants were compared with the concepts that emerged from the second set and so on. The point at which no new concept-relevant insights emerged was considered the point at which saturation was deemed to have been achieved.

## Results

### Participant population

Most participants were aged between 36 and 60 years, 54% of participants were female, and 67% had completed college or higher education. Most participants rated the severity of their nasal obstruction as ‘moderate’ and rated their general health as ‘good’; no participants rated their general health as ‘poor’ or ‘very poor’ (Table 1).

**Table 1:** Participant socio-demographic characteristics

Socio-demographic characteristic	US (n = 12)	Germany (n = 6)	China (n = 6)	Total (n = 24)
**Age (years), n (**%**)**				
18–35	3 (25)	3 (50)	1 (17)	7 (29)
36–60	7 (58)	2 (33)	5 (83)	14 (58)
≥ 61	2 (17)	1 (17)	0 (0)	3 (13)
**Sex n**,** (%)**				
Female	8 (67)	2 (33)	3 (50)	13 (54)
Male	4 (33)	4 (67)	3 (50)	11 (46)
**Race** ^1^				
Non-White	4 (33)		6 (100)^2^	10 (42)
White	8 (67)		0 (0)	8 (33)
**Ethnicity** ^1^				
Non-Hispanic or Latino	10 (83)		6 (100)^3^	16 (67)
Hispanic or Latino (of any race)	2 (17)		0 (0)	3 (8)
**Highest level of education**				
Completed high school or less^4^	5 (42)	1 (17)	2 (33)	8 (33)
Completed college or higher education^5^	7 (58)	5 (83)	4 (67)	16 (67)
**Severity of nasal obstruction**,** n (%)**^6^				
Moderate	9 (75)	6 (100)	4 (67)	19 (79)
Severe	3 (25)	0 (0)	2 (33)	5 (21)
**Diagnosis date**,** n (%)**				
2010 or before	4 (33)	4 (67)	0 (0)	8 (33)
2011–2016	2 (17)	0 (0)	2 (33)	4 (17)
2017 or after	6 (50)	2 (33)	4 (67)	12 (50)
**Diagnosis method**,** n (%)**^7^				
Imaging studies	12 (100)	1 (17)	2 (33)	15 (63)
Nasal endoscopy	6 (50)	1 (17)	4 (67)	11 (46)
General physical examination	3 (25)	4 (67)	1 (17)	8 (33)
Allergy test	0 (0)	3 (50)	2 (33)	5 (21)
Blood test	0 (0)	1 (17)	3 (50)	4 (17)
Other	0 (0)	0 (0)	4 (67)	4 (17)
**General health rating**,** n (%)**				
Fair	3 (25)	0 (0)	6 (100)	9 (38)
Good	6 (50)	6 (100)	0 (0)	12 (50)
Very good	3 (25)	0 (0)	0 (0)	3 (13)

^1^The German Federal Data Protection Act prohibits the collection of ethnic and racial information as part of surveys or studies in Germany, hence there is no ethnic or racial data for the German sample; ^2^all participants in China self-reported their race as Chinese; ^3^all participants in China self-reported their ethnicity as Han Chinese; ^4^defined as elementary, middle, or high school (US), primary or lower secondary education (Germany), or primary or junior secondary education (China); ^5^defined as college, associate, undergraduate, or graduate degree (US), upper secondary education, undergraduate degree, or postgraduate degree (Germany), or senior secondary education, undergraduate degree, or postgraduate degree (China); ^6^as selected on Item 1 of the CRSwNP symptom VRS (‘Please rate your nasal obstruction at its worst over the previous 24 hours’) at the time of screening; participants provided multiple responses

#### Concept elicitation—patient experience of CRSwNP

**Fig. 1:**
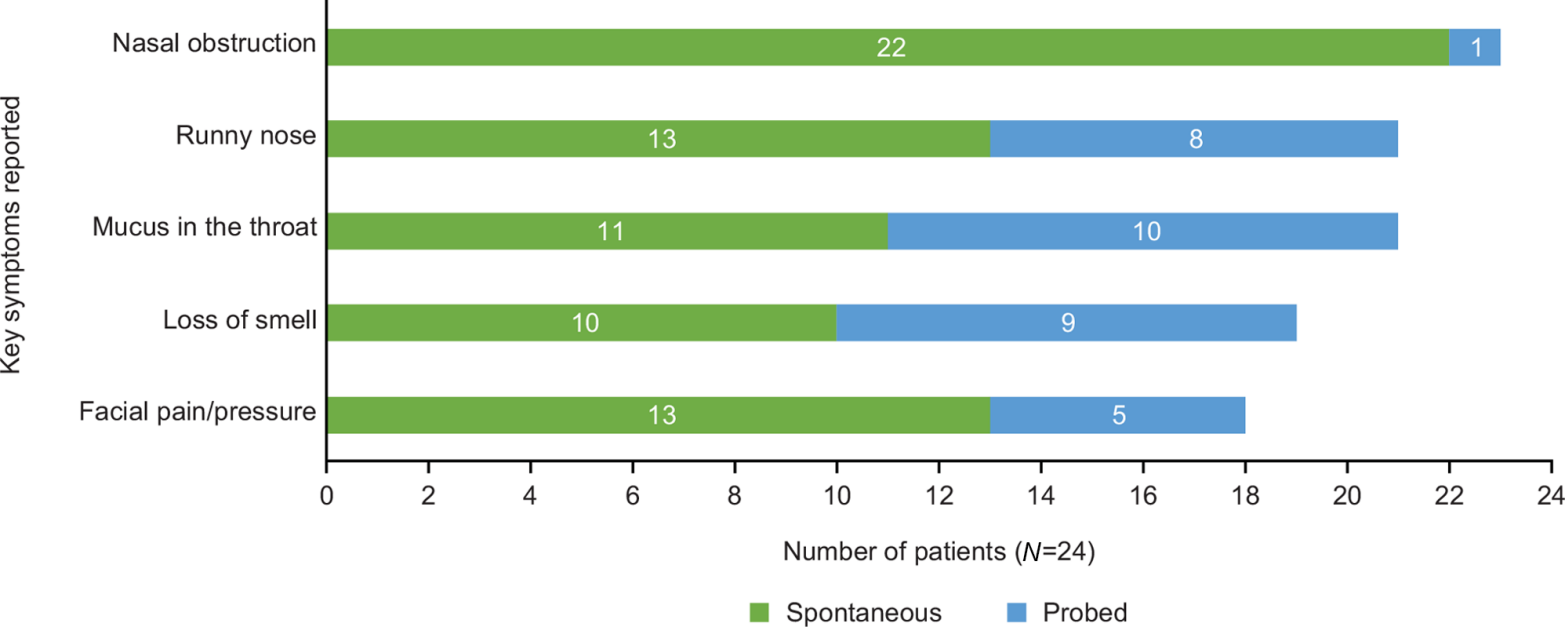
Participant experience of key CRSwNP symptoms

The items assessed by the CRSwNP symptom VRS were the symptoms most frequently reported during the concept elicitation section of the interview (Fig. 1). Based on participant responses on symptom severity and impact on daily activities, most participants reported nasal obstruction (*n* = 13/24, 54%) as the most bothersome symptom. Facial pain or pressure (*n* = 5/24, 21%), loss of smell (*n* = 4/24, 17%), runny nose (*n* = 4/24, 17%), and mucus in the throat (*n* = 3/24, 13%) were also reported by some participants to be the most bothersome symptom they experienced. Other symptoms spontaneously reported by participants included loss of taste (*n* = 6/24, 25%), itchy eyes (*n* = 2/24, 8%), and sneezing (*n* = 2/24, 8%). Examples of participants’ descriptions of the frequency, duration, and severity of their symptoms are included in Supplementary Table 2.

**Fig. 2:**
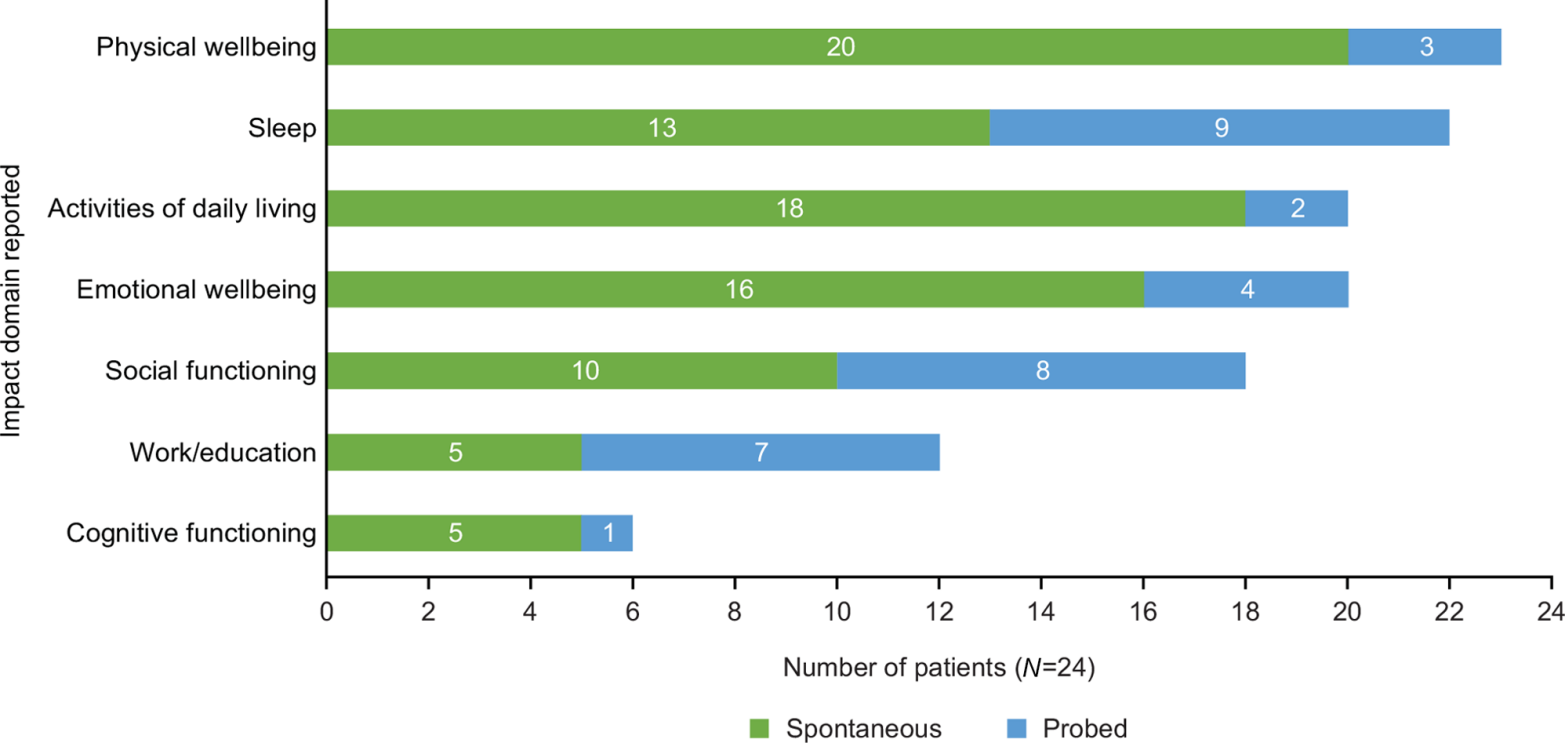
Overview of impact domains reported by participants

All participants (*N* = 24) discussed the impact that CRSwNP symptoms had on their lives, with the most frequently reported domains being physical wellbeing (*n* = 23/24, 96%) and sleep (*n* = 22/24, 92%) (Fig. 2).

All symptoms assessed by the CRSwNP symptom VRS were reported spontaneously for the first time by participants in the first set of interviews. There were additional symptoms reported spontaneously only by a few participants; however, only one of these symptoms (sneezing) was reported spontaneously for the first time in the last set of interviews. Additional symptoms reported are likely allergy symptoms as opposed to NP symptoms; therefore, conceptual saturation was achieved indicating that no further interviews were necessary to fully explore the symptom experience of CRSwNP.

#### Cognitive debriefing of the CRSwNP symptom VRSs, PGI-S, and PGI-C measures

Overall, the VRS items and response options were very well understood by all participants, as 100% of participants were able to select a response that reflected their experience ‘over the past 24 hours’ on four of the five items. The remaining item, nasal obstruction, was understood by 92% of participants; however, it was unclear whether two participants had understood this item: one participant had difficulty selecting an answer as they thought the question was asking about a change in symptoms, and one participant referred to multiple symptoms when describing their selected response. Almost all participants indicated that symptoms assessed by the CRSwNP symptom VRSs were relevant to their experience of CRSwNP; participants had experienced nasal obstruction (100%), runny nose (100%), feeling mucus in the throat (96%), loss of smell (83%), and facial pain/pressure (83%). A small number of participants (n = 4/24, 17%) had not experienced nasal obstruction in the 24 hours prior to the interview and five additional participants (n = 5/24, 21%) indicated that their nasal obstruction was mild in the 24 hours prior to the interview. In addition, some participants indicated that they had never experienced loss of smell (n = 4/24, 17%), facial pain or pressure (n = 4/24, 17%), or the feeling of mucus in the throat (n = 1/24, 4%) due to CRSwNP. Further details are provided in Supplementary Table 3.

Meaningful change was explored at the item level and a within-person one-category improvement was the most frequent level of change reported to be meaningful by participants (Table 2; initial severity scores and meaningful change reported by each participant for each item are shown in Supplementary Fig. 2A–E). Participants who initially reported severe symptoms were more likely to consider a two- or three-category change as meaningful than those with moderate symptoms Supplementary Fig. 2A–E). Participant rationales for perceiving a one- or two-category improvement levels as meaningful to them are summarized in Supplementary Table 4. For the ‘nasal obstruction’ item, improved breathing (*n* = 8) was the most common reason participants considered a one-category change to be meaningful; for the ‘runny nose’ item, the most common reasons were reduced frequency in experiencing the symptom (*n* = 9) and reduced need to blow or wipe their nose (*n* = 6). For the ‘feeling of mucus in the throat’ and ‘loss of smell’ items, the most common reasons were reduced coughing or clearing of the throat (*n* = 6) and an ability to distinguish scents more easily (*n* = 5), respectively. Finally, for the ‘facial pain or pressure’ item, the most common reason for considering a one-category improvement meaningful was a reduction in pain (*n* = 4).

**Table 2:** Meaningful change for CRSwNP symptom VRSs at the item level

Meaningful category change^1^	Item 1: Nasal obstruction (N = 24)	Item 2: Runny nose (N = 24)	Item 3: Mucus in the throat (N = 23)	Item 4: Loss of smell (N = 20)	Item 5: Facial pain and pressure (N = 20)
1 category	n = 19	n = 21	n = 17	n = 14	n = 14
2 categories	n = 5	n = 3	n = 5	n = 6	n = 6
3 categories	n = 0	n = 0	n = 1	n = 0	n = 0

^1^Response options on the CRSwNP symptom VRSs from ‘No symptoms’ to ‘Severe symptoms’

Participants generally provided positive feedback on the CRSwNP symptom VRS items and response options; they reported that the instrument took an acceptable amount of time to complete, it was easy to select a response for each item, and 92% (*n* = 22/24) reported that it would be feasible for them to complete the CRSwNP symptom VRSs every day for several weeks if required to do so during a clinical trial.

#### Participant understanding and meaningful change of the PGI-S and PGI-C

The PGI-S and PGI-C item and response options were understood by all participants (*N* = 24). Most participants (*n* = 19/24, 79%) considered a within-person one-category improvement in PGI-S to be meaningful to them, regardless of symptom severity. For the PGI-C, 58% (*n* = 14/24) of participants considered a within-person improvement of ‘a little better’ versus the start of a trial to be meaningful to them.

## Discussion

This qualitive interview study supports the use of the five CRSwNP symptom VRS items for the assessment of symptoms in patients with moderate-to-severe CRSwNP. The CRSwNP symptom VRS items have been shown to comprehensively assess the symptoms most relevant to patients with CRSwNP. Key symptoms reported spontaneously by study participants aligned with the five items selected for inclusion in a novel CRSwNP symptom VRS measure. The five CRSwNP symptom VRS items were relevant to the majority of participants’ experience of living with CRSwNP and its impact on daily activities. Participants demonstrated a good understanding of the VRS items, were satisfied with the length of time taken to complete the measure and deemed the daily assessment and recall period of 24 h appropriate and feasible to complete in a clinical trial setting. Conceptual relevance findings are consistent with prior qualitative research conducted to support content validity of the overall CRSwNP symptom VAS [9, 17].

While this study did not compare CRSwNP symptom VAS and VRS directly, it demonstrated that the four-level VRSs recommended in FDA guidance [11] provide a valid and reliable alternative measurement option.This is further supported by results of a recent study in participants with CRS, who indicated that the use of a 4-point response scale (i.e., ‘None’, ‘Mild’, ‘Moderate’, and ‘Severe’) to assess CRS symptom severity is easiest to understand and easy to use (relative to a VAS and five Likert scales ranging from four to eight items) [18]. The use of five or fewer response categories is also consistent with recommendations for the selection of response scales for PRO instruments [19].

A strength of the study was the recruitment of participants who met eligibility criteria aligned with ongoing Phase III trials in CRSwNP, including ANCHOR-1 (NCT05274750) and ANCHOR-2 (NCT05281523), two replicate randomized, double-blind, placebo-controlled studies investigating the efficacy and safety of depemokimab in patients with CRSwNP. Participants were required to have moderate-to-severe nasal obstruction and a history of previous surgery or SCS use; this representative sample ensures generalizability of the findings to the target population for new treatments for patients with moderate-to-severe CRSwNP. The overall sample size (*N* = 24) and that for the US population (*n* = 12), although small, were considered appropriate for the aims of exploring content validity of a PRO measure and for assessing the comprehensiveness of the items and identifying potential problems [20]. A further strength of the concept elicitation section of the study was the use of both broad, open-ended questions and focused probes to explore concepts not mentioned spontaneously or requiring additional clarification. For the cognitive debriefing section, comprehensive interview materials were provided by email. Robust quality control measures were in place for data analysis and data interpretation.

A potential limitation of the study is that it was conducted retrospectively to evaluate the content validity of existing questionnaires, which may introduce bias. This was mitigated to some extent by ensuring participants did not see the questionnaires until after the concept elicitation portion of the interview. Additionally, while this study provided a qualitative evaluation of the within-person improvement that constitutes meaningful change, quantitative assessment (such as with anchor-based methods) is still necessary [21]. In particular, further assessment of PGI-C in the context of a clinical trial is required, as participants in this study were only rating hypothetical changes. A further consideration is the use of a 4-week recall period with the PGI-C and PGI-S measures as a long recall period could lead to a potential recall bias. Another potential limitation is the educational imbalance among participants; the proportion of who had completed high school or less was lower than those who had completed college or higher education, particularly in Germany, where only one out of six participants had completed high school or less. The within-person improvement that would constitute meaningful change at the item level was consistent across CRSwNP symptom VRS items, with a one-category improvement most frequently reported as meaningful. Similarly, a one-category improvement in PGI-S was considered meaningful to participants, while an improvement of ‘a little better’ from baseline was considered meaningful for the PGI-C. When interpreting the meaningful change results, it should be noted that some participants had only experienced mild symptoms or no symptoms in the 24 h prior to the interview; as such, these results are considered preliminary and should be supplemented by more traditional anchor- and distribution-based quantitative estimates of meaningful change. For some participants, the self-reported severity of nasal obstruction between screening and interview did not align, and the experience of mild or no symptoms by some participants may have affected the interpretation of meaningful change scores. However, it should be noted that the CRSwNP VRSs are intended for daily use with scores averaged across a 7-day period to generate a mean score per item, which would allow for capturing the day-to-day variance of symptoms without undue weight being given to a single day.

Further psychometric evaluation may be required to evaluate the reliability, validity, and sensitivity to change of CRSwNP symptom VRSs and their appropriateness for use in clinical trials, since they use ordinal-level response options rather than the interval-level response options used for the CRSwNP symptom VAS.

## Conclusions

The five CRSwNP symptom VRSs assess the most relevant and bothersome symptoms experienced by participants with moderate-to-severe CRSwNP and the five VRS items are well understood by participants. The study provides preliminary insights into clinically meaningful improvements in symptom severity and the overall findings support the content validity of the CRSwNP symptom VRSs, as well as the PGI-S and PGI-C measures, among a diverse sample of participants in the US, Germany, and China.

## Electronic Supplementary Material

Below is the link to the electronic supplementary material.

Supplementary Material 1

## Data Availability

For requests for access to anonymized subject level data, please contact the corresponding author.

## Abbreviations

CRSwNP: Chronic rhinosinusitis with nasal polyps
DSL: Data Security Law
EMA: European Medicines Agency
FDA: Food and Drug Administration
GDPR: General Data Protection Regulation
HRQoL: Health-related quality of life
IRB: Institutional Review Board
PGI-C: Patient Global Impression of Change
PGI-S: Patient Global Impression of Severity
PIPL: Personal Information Protection Law
PRO: Patient-reported outcome
SCS: Systemic corticosteroids
VAS: Visual analog scale
VRS: Verbal response scale
