# Supplementary material for: Chronic rhinosinusitis with nasal polyps (CRSwNP) symptom verbal response scales: content validity testing for use in adults with CRSwNP
Source: J Patient Rep Outcomes. 2024 Dec 20;8:152. doi: 10.1186/s41687-024-00827-4 (PMC11662121; doi:10.1186/s41687-024-00827-4)
Supplement: Supplementary file 1 — Supplementary Material 1 [file 41687_2024_827_MOESM1_ESM.pdf]

Supplementary Material

Supplementary Methods

Interviews in the US were conducted by two trained and experienced qualitative interviewers from Adelphi Values (RS and SW). Both interviewers participated in a comprehensive briefing and mock interview (e.g., role-play session) with the project lead (KF) prior to conducting any interviews to ensure they were fully familiar with the interview guide and understood what information should be obtained from the interview according to the study objectives.

Interviews in Germany and China were conducted in the local language by a trained and experienced qualitative interviewer (educated to a degree level or above) in each country. Adelphi Values provided interviewers with training in English, which included a breakdown of the content of the interview guide and the interviewing process. Following the training, Adelphi Values were available to answer any questions interviewers may have had to ensure the study objectives were met.

Supplementary Table 1. Sampling quotas for participant socio-demographic characteristics

**Participant characteristics**	**Sampling quotas (N=24)**		
	**US (n=12)**	**Germany (n=6)**	**China (n=6)**
**Age (years)**			
18–35	≥2	≥1	≥1
36–60	≥2	≥1	≥1
≥61	≥2	≥1	≥1
**Gender**			
Male	≥4	≥2	≥2
Female	≥4	≥2	≥2
**Race**[1]			
White	≥3	N/A	N/A
Non-white	≥3	N/A	N/A
**Ethnicity**[1]			
Hispanic or Latino (of any race)	≥3	N/A	N/A
Non-Hispanic or Latino	≥3	N/A	N/A
**Education level**			
Completed high school or less	≥4	≥2	≥2
Completed college or higher education	≥4	≥2	≥2

N/A denotes a participant characteristic that is not applicable to obtain/target based on the country's culture.

[1]The German Federal Data Protection Act prohibits the collection of ethnic and racial information as part of surveys or studies in Germany, hence there is no ethnic or racial data for the German sample. Chinese and Han Chinese are the largest racial and ethnicity groups in China (the majority of Chinese individuals identify as these characteristics); therefore, no quotas were implemented for these characteristics for the Chinese sample.

Supplementary Table 2. Overview of key CRSwNP symptoms reported by participants

Description of participant experiences of symptom[1]	Example supporting quotes
**Nasal obstruction (n=23/24, 96%)**	
Participants reported experiencing nasal obstruction:<br>• Daily to yearly in terms of frequency.<br>• Constantly in terms of duration (when experienced).<br>• Moderately or severely in terms of severity.	*"It starts out where you can't breathe a little bit and then you're to the point where you have to get them removed. Your nose is completely stuffed up."*<br>*"…I'm always restricted in terms of breathing through my nose… it's actually really severe again. And now it's just constant… my nose is continuously blocked."*
**Runny nose (n=21/24, 88%)**	
Participants reported experiencing a runny nose:<br>• Daily to yearly in terms of frequency.<br>• Constantly or momentarily during the day in terms of duration (when experienced).<br>• Mildly or severely in terms of severity.	*"I sniff so often that I don't even realize I do it until somebody asks me if I'm sick or if I feel okay… I always have like a stuffy or running nose…"*<br>*"As soon as I wake up, usually I already have a slightly runny nose. And over the course of the day, it can happen that I often use two whole packets of tissues for it in total…"*
**Mucus in the throat (n=21/24, 88%)**	
Participants reported experiencing a feeling of mucus in their throat:<br>• Daily to yearly in terms of frequency.<br>• Constant or momentarily during the day in terms of duration (when experienced).<br>• Mildly or moderately in terms of severity.	*"…a lot of phlegm collects in my throat, and I have to work really hard on [relieving] that…Every 2 days I would actually feel that a bit more had accumulated there… I clear my throat a lot and try to get as much phlegm out of my throat as possible."*<br>*"…I seem to have like a lot of mucus… that is very uncomfortable in that, um, I'm clearing my throat all the time."*
**Loss of smell (n=19/24, 79%)**	
Participants reported experiencing loss of smell:<br>• Daily to yearly in terms of frequency.<br>• Constantly or momentarily during the day in terms of duration (when experienced).<br>• Mildly, moderately, or severely in terms of severity.	*"I haven't smelled or tasted properly in the past like 3 or 4 years… there are sometimes when I can't. I literally cannot detect a smell."*<br>*"Um, total loss of smell I would say happens maybe once or twice a month. Um, but the feeling of having a lessened sense of smell is all the time."*

**Facial pain or pressure (n=18/24, 75%)**	
Participants reported experiencing facial pain or pressure:<br>• Daily to yearly in terms of frequency.<br>• Constantly in terms of duration (when experienced).<br>• Moderately in terms of severity.	*“Definitely pain in my face, or this pressure that I feel around my sinuses. Feeling as if something was pressing on them all the time…”*<br>*“…The pressure seems to always be there. And, um, it’s, it’s not a pleasant feeling… it’s very uncomfortable… all day… Every day.”*

[1]The following criteria were used to describe participant experiences of symptoms in relation to severity: Mild: Symptom can be well-managed on a daily basis (without surgery or visiting a medical clinic), with no significant impact on daily life and activities; Moderate: Symptom requires at-home coping method(s) to manage and relieve it, may occasionally impact daily life but can be mitigated; symptom affects the participants some of the time; Severe: Symptom occasionally requires surgery/medical attention to relieve, and at-home coping methods do not help despite attempts to control; symptom is consistently impacting daily life.

Supplementary Table 3. Participant understanding and relevance of CRSwNP symptom VRS items

Understanding	Relevance
**Item 1: 'Please rate your nasal obstruction at its worst over the previous 24 hours' (no symptoms, mild symptoms, moderate symptoms, severe symptoms)**	
• 22/24 (92%) participants demonstrated an understanding of both the item and response options, as they were able to select an appropriate response that represented their experience of nasal obstruction over the previous 24 hours.<br>*"Um, well the question is specific in saying at its worst over the past 24 hours. And as I mentioned before, uh, for me, the inflammation peaks in the morning and then the evening. So, in the evenings I would certainly at a minimum rate it as a moderate condition. Um, there are times when it's severe, but, you know, thinking about the last 24 hours for myself, I would say that a moderate, uh, aligns perfectly with what I've experienced."*<br>• 1/24 (4%) participant had difficulty selecting an answer for the item as they thought the question was asking about a change in symptoms. Of note, this participant did understand the remaining items as intended.<br>*"I was reading the question in a way that I thought it would be, it would be like in the last 24 hours have I experienced any changes in my symptoms."*	• 24/24 (100%) participants indicated that nasal obstruction was relevant to their experience of CRSwNP. Of note, one participant reported not experiencing nasal obstruction in relation to CRSwNP in the CE section but did report experiencing this during CD.<br>• 20/24 participants reported that they experienced nasal obstruction over the previous 24 hours.<br>• *"The worst within 24 hours. I'm in moderate condition right now."* **Interviewer:** ***"Moderate symptoms?"*** *"Yes, my current condition is moderate within 24 hours."* **Interviewer:** ***"Okay, so do you think this question is meaningful to you? Is it related to your nasal polyps?"*** *"This is just to understand my condition, that is, my worst case of nasal congestion. It's kind of related."* **Interviewer:** ***"Is this because you also have nasal congestion, you have the symptom of nasal congestion from nasal polyps?"*** *"Right."*<br>• 4/24 (17%) participants reported that they do experience nasal obstruction due to CRSwNP but had not experienced the symptom during the 24 hours prior to the interview. Of note, in the CE portion of the interview, three of these participants spontaneously reported nasal obstruction as a symptom of CRSwNP. The remaining participant noted that they experience the symptom, but they did not attribute it to CRSwNP (no further information was provided).<br>*"I just had them [nasal polyps] removed about 3 months ago, so no symptoms…[before the surgery] well 24 hours a day. It didn't—it never goes away. It just gets worse…It starts out mild and then it ends up severe."*

Understanding	Relevance
• 1/24 (4%) participant referred to multiple symptoms, as opposed to nasal obstruction only, when describing their selected response so it is not clear if they understood the item or not. *"I would probably [select] moderate symptoms [because] I definitely wake up every day with symptoms as far as, um, some days it's more mucus and more, um, post-nasal drip. And, um, the pressure in my face is probably moderate every day."*	
**Item 2: 'Please rate your runny nose at its worst over the previous 24 hours' (no symptoms, mild symptoms, moderate symptoms, severe symptoms)**	
• 24/24 (100%) participants demonstrated an understanding of the item and response options, as they were able to select an appropriate response that represented their experience of runny nose in the previous 24 hours. *"I didn't have that of course, because I had "no symptoms" in the last 24 hours."*	• 24/24 (100%) participants reported that runny nose was relevant to their experience of CRSwNP.<br>• 18/24 participants reported that they experienced a runny nose in the previous 24 hours.<br>• *"I would say moderate symptoms. Because it's actually constant the whole time, I blow my nose and I still feel like there's something there, but nothing more comes out. And I still have this constant running. It just doesn't get any better."*<br>• 6/24 (25%) participants reported that they do experience a runny nose due to CRSwNP but had not experienced the symptom during the 24 hours prior to the interview. *"I don't really have a runny nose because my mucus is thick. So, I suppose I would choose no symptoms…there have been occasions where it's severe. You know, 9.9 out of 10 days [the mucus is] going to be very thick and gluey. Um, but there are—there have been times where, you know, for whatever reason, um, I have a day where it's just very loose and runny. Um, and on those very rare occasions, I would put it at a severe level, but they are very rare."*

Understanding	Relevance
**Item 3: 'Please rate your feeling of mucus in the throat at its worst over the previous 24 hours' (no symptoms, mild symptoms, moderate symptoms, severe symptoms)**	
• 24/24 (100%) participants demonstrated an understanding of both the item and response options, as they were able to select an appropriate response that represented their experience of feeling of mucus in the throat in the previous 24 hours.<br>*"I would answer it mild symptoms…Severe to me sounds like, you know, you've lost your voice almost and you have trouble talking. That's what I envision severe to be. I just sort of have this feeling of like needing to clear my throat frequently."*<br>*"For me, [the feeling of mucus in the throat is] like fairly mild because I haven't spent like the last 24 hours hawking loogies [coughing up mucus] like non-stop. But the fact that I've had post-nasal drip, like for someone else that might be moderate."*	• 23/24 (96%) participants reported that a feeling of mucus in the throat was relevant to their experience of CRSwNP.<br>• 19/23 participants reported that they had experienced mucus in the throat in the last 24 hours.<br>*"I would perhaps say mild symptoms. That would have been yesterday evening, I think."*<br>• 4/23 participants reported that they do experience mucus in the throat in relation to CRSwNP but had not experienced the symptom during the 24 hours prior to the interview.<br>**Interviewer: *"The only time you felt mucus in your throat was before you were hospitalized. That was last year, right?"*** *"Yes."* **Interviewer: *"When you go back to your symptoms at that time, which degree would you choose?"*** *"Severe symptoms."* **Interviewer: *"Please recall the sense of mucus in the throat at that time and describe what you experienced or what happened."*** *"At that time, I was always coughing, coughing, and it felt like it was coughing up from the lungs. In fact, it was sticking to the throat. I always wanted to cough and blow my nose, then coughed up a mouthful of sputum."*<br>• 1/24 (4%) participant reported never having experienced a feeling of mucus in the throat in relation to CRSwNP.<br>*"'Please evaluate the severity of mucus feeling in your throat in the past 24 hours.'"* **Interviewer: *"Mucus feeling in throat. Which answer would you choose?"*** *"I choose 'no'."* **Interviewer: *"No symptoms, okay…You said that you have not experienced this condition, so it has never happened to you, right?"*** *"It didn't happen to me."* **Interviewer: *"It didn't happen to you, okay. So, you said there are no symptoms. You just said that you never have a serious feeling of mucus in the throat."*** *"Not in my memory."*
**Item 4: 'Please rate your loss of smell at its worst over the previous 24 hours' (no symptoms, mild symptoms, moderate symptoms, severe symptoms)**	
• 24/24 (100%) participants demonstrated an understanding of both the item and response options, as they were able to select an appropriate response that represented their	• 20/24 (83%) participants reported that loss of smell was relevant to their experience of CRSwNP.<br>• 2/20 participants reported that they had experienced loss of smell in the last 24 hours.

Understanding	Relevance
experience of loss of smell in the previous 24 hours.<br>*"I would say mild symptoms because when it—with my polyps this time, I don't really have the loss of smell, um, constantly. If I have really bad congestion that day, I'll have the loss of smell. But, um, it's actually not that bad as, as it was last time, so I'm just going to say mild."* (US12-F32-LOW-SEV)<br>*"Um, I've had no loss of smell over the past 24 hours, so no symptoms."*	*"My nose smelt something 3 weeks ago, but I did not know the reason."* **Interviewer:** ***"How did you feel at that time?"*** *"I felt good and was very happy, but I cannot smell again in these days. I asked my doctor the night before, and he told me my nasal polyp might have regenerated again over time, or it blocked my olfactory organ."*<br>• 8/20 participants reported that they do experience loss of smell in relation to CRSwNP but had not experienced the symptom during the 24 hours prior to the interview.<br>*"I've had no loss of smell over the past 24 hours, so no symptoms."* **Interviewer:** ***"Um, so how often would you say you experience loss of smell?"*** *"Maybe three, three or four times a year when I'm super blocked up and stuffy."*<br>• 4/24 (17%) participants reported that they had never experienced loss of smell in relation to CRSwNP.<br>*"Um, I really don't have any [loss of smell], so that would be no symptoms."* **Interviewer:** ***"And, um, so is this something that you've ever experienced in the past?"*** *"I have not."*

Understanding	Relevance
**Item 5: ‘Please rate your facial pain or pressure at its worst over the previous 24 hours’ (no symptoms, mild symptoms, moderate symptoms, severe symptoms)**	
• 24/24 (100%) participants demonstrated an understanding of both the item and response options, as they were able to select an appropriate response that represented their experience of facial pain or pressure in the previous 24 hours.<br>*“I woke up with a headache this morning. Although it was mild or moderate, more like mild. It can be much worse sometimes…[it feels like] kind of a warm feeling, as if somebody were actually pressing on my eyes and on my nose area from the inside the whole time. It’s really unpleasant. A certain type of headache.”*<br>*“I'm going to say I have that moderate because I have had it pretty bad for the past 24 hours. Um, right, uh, right outside of my nose, I've had a lot of pressure there. Um, so I'm going to say moderate for that one.”*	• 20/24 (83%) participants reported that facial pain or pressure was relevant to their experience of CRSwNP.<br>• 15/20 participants reported that they experienced facial pain or pressure in the last 24 hours.<br>*“I would say moderate symptoms…I don’t know if it's because I'm having this conversation right now or not, but I'm aware that there's a lot of facial pain and pressure right now.”*<br>• 5/20 participants reported that they do experience facial pain or pressure in relation to CRSwNP but had not experienced the symptom during the 24 hours prior to the interview.<br>*“In the last 24 hours, I had no pain or feeling of pressure in my face, fortunately… [but I do get it] four or five times a month.”*<br>• 4/24 (17%) participants reported that they had never experienced facial pain or pressure due to CRSwNP.<br>*“I would have to say no symptoms… [I did get it] sometimes when I was diving, but that has nothing to do with the nasal polyps.”*

Supplementary Table 4. Participant rationale for why the category improvement identified for VRS items would be meaningful to them

One-category improvement	Two-category improvement
**Item 1: Nasal obstruction (N=24)**	
**n=19**	**n=5**
• Improved breathing (n=8)<br>• Reduced amount of obstruction (n=3)<br>• Reduced frequency of runny nose/blowing nose (n=2)<br>• Better sense of smell (n=1)<br>• Better sense of taste (n=1)<br>• Reduced facial pressure (n=1)<br>• Improved sleep (n=1)<br>• Improved work activities (n=1)	• Improved breathing (n=3)<br>• Reduced facial pressure (n=1)<br>• Reduced mucus (n=1)<br>• Improved hearing (n=1)
**Item 2: Runny nose (N=24)**	
**n=21**	**n=3**
• Symptom experienced less often (n=9)<br>• Blow/wipe their nose less often (n=6)<br>• Reduced snoring (n=1)<br>• Less frequent mucus (n=1)<br>• Reduced nose soreness (blowing nose less; n=1)<br>• Reduced impact to work activities (sniffing, wiping nose at work; n=1)	• No longer required to carry tissues to blow their nose (n=1)
**Item 3: Feeling of mucus in the throat (n=23)**	
**n=17**	**n=5**
• Reduction in coughing or clearing the throat (n=6)<br>• Less mucus in the throat (n=5)<br>• Feeling more comfortable overall (n=4)<br>• Increased ability to expel mucus (n=2)<br>• Improved breathing (n=1)	• Less mucus in the throat (n=3)<br>• Reduction in coughing or clearing the throat (n=1)<br>• Reduced annoyance of the symptom (n=1)

One-category improvement	Two-category improvement
**Item 4; Loss of smell (n=20)**	
**n=14**	**n=6**
• Able to distinguish scents more easily, such as out of date food (n=5)<br>• Improved sense of taste and enjoyment of food (n=3)	• Improved sense of taste and enjoyment of food (n=2)<br>• Able to distinguish scents more easily e.g., gas leaks (n=2)<br>• Smell is considered an important sense (n=1)
**Item 5: Facial pain or pressure (n=20)**	
**n=14**	**n=6**
• Reduction in pain (n=4) or pressure (n=1)<br>• Improved breathing (n=2)<br>• Less distracted by the symptom (n=2) and able to focus more on work or family (n=1)<br>• Rely less on medication to treat the symptom (n=2)<br>• Improvements in headaches and runny nose (n=1)<br>• Improved sleep (n=1)<br>• Able to wear glasses without discomfort (n=1)<br>• Feeling more comfortable (n=1)	• Reduction in pain (n=3) would allow the participant to rest even if the symptom was not completely absent

Supplementary Figure 1. Participant recruitment procedure overview

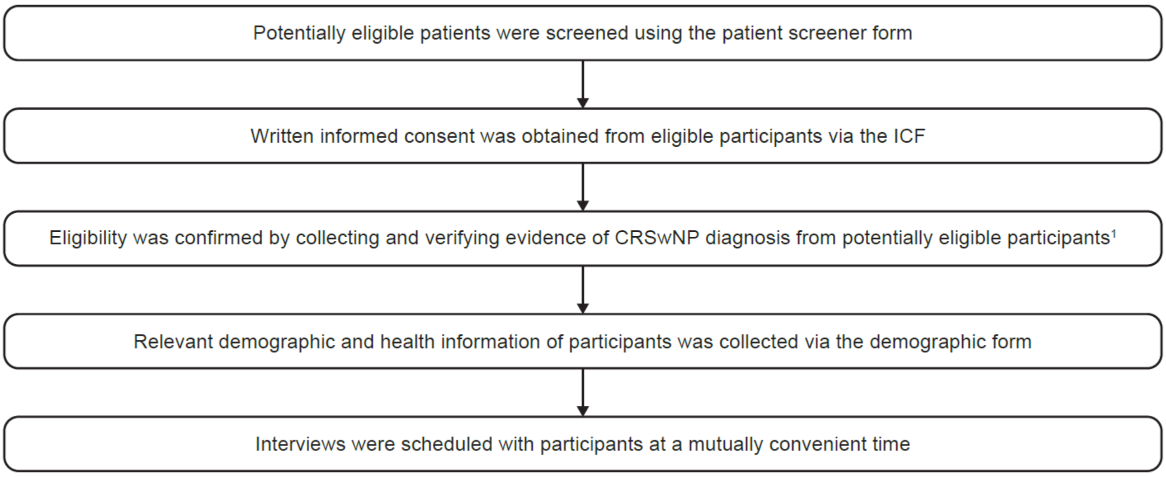

[1]For data protection purposes, no personal identifiable information for participants from China was shared with Adelphi Values; the recruitment agency reviewed participants' evidence of CRSwNP diagnosis and participant screener forms and confirmed participants' eligibility with Adelphi Values.

ICF, informed consent form.

Supplementary Figure 2. Initial VRS item scores and meaningful within-person improvement scores reported by each participant used for assessment of meaningful change in A. nasal obstruction; B. runny nose; C. mucus in the throat; D. loss of smell; E. facial pain/pressure

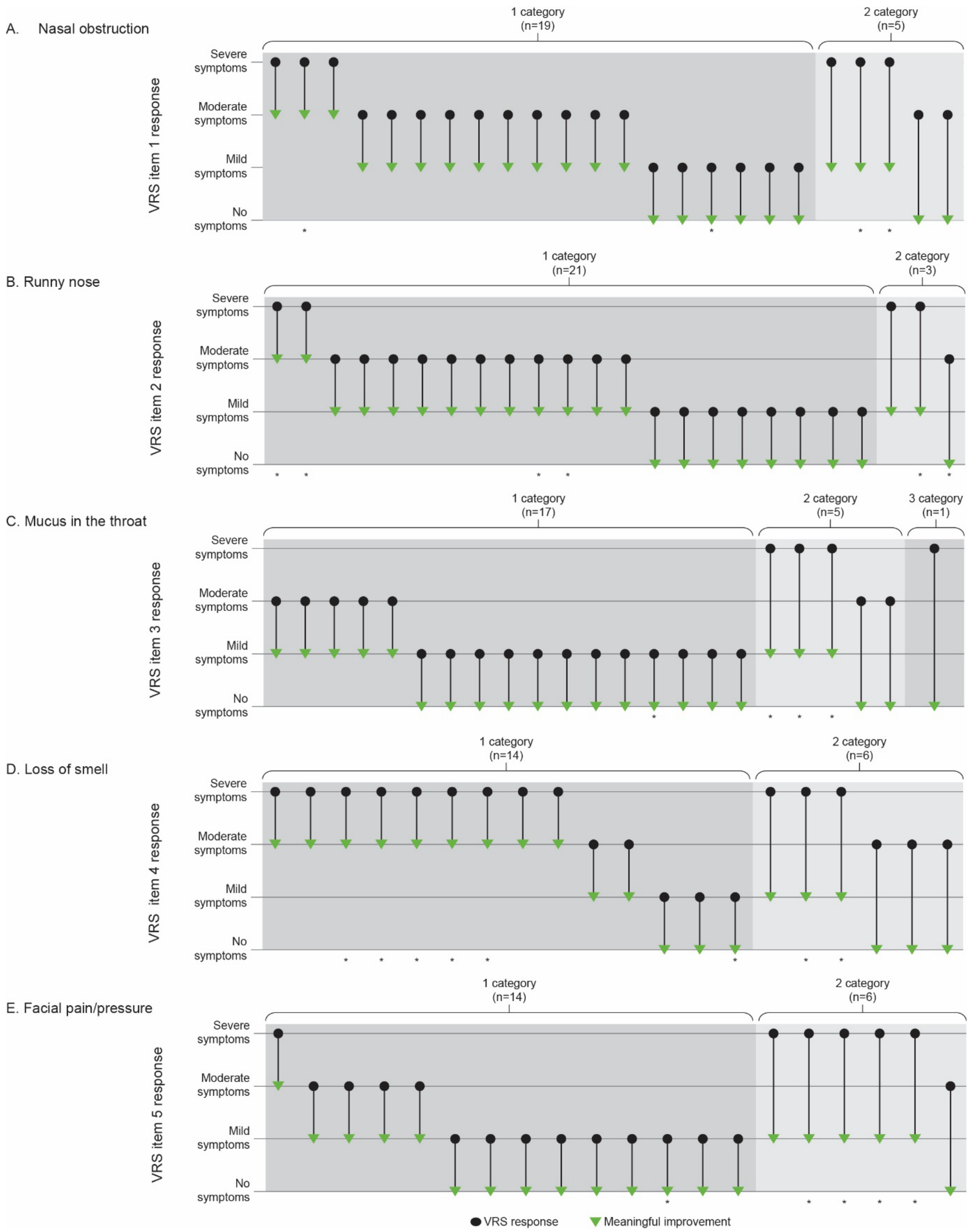

*Based on hypothetical responses as the symptom was not experienced within the timeframe specified in the item.
